# Elastic, electronic and optical properties of new 2D and 3D boron nitrides

**DOI:** 10.1038/s41598-020-64866-9

**Published:** 2020-05-12

**Authors:** Huayue Mei, Yuhan Zhong, Dafang He, Xue Du, Chunmei Li, Nanpu Cheng

**Affiliations:** School of Materials and Energy, Southwest University, Chongqing, 400715 China

**Keywords:** Theory and computation, Condensed-matter physics

## Abstract

The current work investigates a novel three-dimensional boron nitride called bulk B_4_N_4_ and its corresponding two-dimensional monolayer B_4_N_4_ based on the first-principles of density functional theory. The phonon spectra prove that bulk B_4_N_4_ and monolayer B_4_N_4_ are dynamically stable. The molecular dynamics simulations verify that bulk B_4_N_4_ and monolayer B_4_N_4_ have excellent thermal stability of withstanding temperature up to 1000 K. The calculated elastic constants state that bulk B_4_N_4_ and monolayer B_4_N_4_ are mechanically stable, and bulk B_4_N_4_ has strong anisotropy. The theoretically obtained electronic structures reveal that bulk B_4_N_4_ is an indirect band-gap semiconductor with a band gap of 5.4 eV, while monolayer B_4_N_4_ has a direct band gap of 6.1 eV. The valence band maximum is mainly contributed from B-2*p* and N-2*p* orbits, and the conduction band minimum mainly derives from B-2*p* orbits. The electron transitions from occupied N-2*p* states to empty B-2*p* states play important roles in the dielectric functions of bulk B_4_N_4_ and monolayer B_4_N_4_. The newly proposed monolayer B_4_N_4_ is a potential candidate for designing optoelectronic devices such as transparent electrodes due to its high transmissivity.

## Introduction

In recent years, the physicochemical properties of boron nitrides in different crystal structures are widely studied^1^. Besides the work on the four common boron nitrides, namely hexagonal boron nitride (h-BN)^2^, rhombohedral boron nitride (r-BN)^3^, cubic boron nitride (c-BN)^4^ and wurtzite boron nitride (w-BN)^5^, researchers have also devoted themselves to the preparations and properties of BN nanosheets^6^. Low dimensional BN nanomaterials, such as h-BN, are famous for their excellent chemical inertness and thermal stability^7^. L. Li *et al*. studied the effect of thicknesses of h-BN on the electric field screening by electrostatic force microscopy (EFM) and theoretical calculations, and found that h-BN is excellent dielectric substrate to support two-dimensional (2D) nanomaterials such as graphene, and MoS_2_^8^. J. Li *et al*. calculated the geometrical evolutions, electronic structures and magnetic features of (111) plane-hydrogenated c-BN nanosheets, and pointed out that they can be applied to novel high-performance optoelectronic, spintronic and electronic nanodevices^9^. S. Lin *et al*. investigated the metal-free Si-doped h-BN nanosheets (Si-BNNS), and stated that they are highly stable and active CO oxidation catalysts^10^. Further, the amorphous boron nitrite with a band gap of 2.0 eV^11^, Al-doped boron nitride nanosheets with mono-vacancy defects for adsorbing formaldehyde^12^, and hydrogen storage properties of oxygen-doped boron nitride nanosheets were also studied^13^.

New structures distinct from the common boron nitrides mentioned above are also the goals explored constantly by researchers. K. Doll *et al*. reported ten two-dimensional layered and three-dimensional (3D) network boron nitrides through *ab initio* simulated annealing^14^. X. Li *et al*. predicted three single-layered boron nitride sheets with *sp*^2^ and *sp* hybrid chemical bonds, and these single-layered boron nitride sheets have promising applications in nanoscale electronic and optoelectronic devices by tuning their band gaps through changing the *sp*-bond lengths in the boron nitride sheets^15^.

In the current work, we investigated a novel 3D monoclinic boron nitride (bulk B_4_N_4_) which was ever proposed by K. Doll^14^ through the CRYSTAL06 code based on local Gaussian-type orbitals. Bulk B_4_N_4_ consists of BN monolayers connected by van der Waals force, and there is still lack of researches on its properties. Further, the special monoclinic B_4_N_4_ structure with neighboring atomic layers bonded by van der Waals force inspired us to build its corresponding one-layered sheet (monolayer B_4_N_4_). Their structural stabilities were verified by the calculations of phonon spectra, molecular dynamics (MD) simulations and elastic constants. Moreover, the mechanical properties, electronic structures and optical properties of bulk B_4_N_4_ and monolayer B_4_N_4_ were calculated to reveal their physical properties in detail, which is beneficial to future experimental studies and potential performances.

## Theoretical Methods and Models

The current calculations were performed by Cambridge Sequential Total Energy Package (CASTEP) which is a quantum computing program based on density functional theory^16^. The generalized gradient approximation (GGA) proposed by Perdew-Burke-Ernzerhof (PBE)^17^ was adopted to describe the electronic exchange-correlation term, and the ultrasoft pseudopotential was applied to depict the interaction between valence electrons and ion core during the calculations of geometry optimization, phonon spectra and mechanical properties. We used the hybrid functional PBE0 and the norm conserving pseudopotentials to calculate the electronic structures and optical properties of bulk B_4_N_4_ and monolayer B_4_N_4_, because the GGA-PBE level often underestimates the band gaps of semiconductors due to the discontinuity of exchange correlation energy^18,19^. The energy cutoffs for the plane wave basis expansion were respectively 280 and 770 eV corresponding to the ultrasoft pseudopotentials and the norm conserving pseudopotentials. The k-points were set to 5 × 3 × 3 and 3 × 3 × 1 Monkhorst–Pack grids for bulk B_4_N_4_ and monolayer B_4_N_4_, respectively. A vacuum space of 20 Å was chosen to avoid the interlayer interaction for monolayer B_4_N_4_. The MD simulations in 2 × 2 × 1 supercells of bulk B_4_N_4_ and monolayer B_4_N_4_ within the canonical ensemble (NVT) were calculated at room temperature (300 K) and high temperature (1000 K) for 5 ps with a time step of 1 fs. The convergence thresholds of geometry optimization adopting the Broyden–Flecher–Goldfarb–Shanno (BFGS) method ^20^ satisfied the following conditions: the total energy was less than 5 × 10^−6^ eV/atom, the ionic force was within 0.01 eV/Å, the stress component was below 0.02 GPa, and the atomic displacement value was less than 5 × 10^−4^ Å. The valence electrons were B 2*s*^2^ 2*p*^1^ and N 2*s*^2^ 2*p*^3^ during all the calculations.

## Results and Discussions

**Figure 1:**
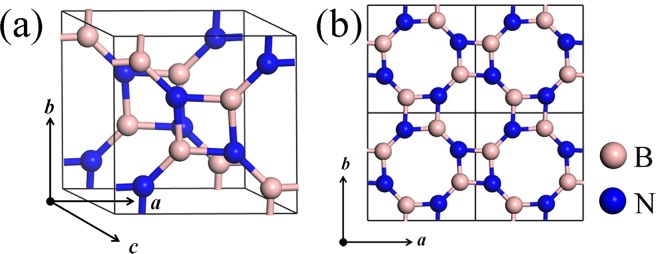
Crystal structures of (**a**) bulk B_4_N_4_ and (**b**) monolayer B_4_N_4_ (2 × 2 × 1 super cell).

Belonging to the monoclinic system, the unit cell of bulk B_4_N_4_ with P21/c space group (No.14) includes four B atoms and four N atoms, seeing Fig. 1(a). The optimized lattice parameters of bulk B_4_N_4_ are *a* = 4.9199 Å, *b* = 4.9294 Å, *c* = 3.3738 Å, *α* = 115.11° and *β* = *γ* = 90°. Interestingly, B_4_N_4_ crystal consists of BN monolayers connected by van der Waals force along the *c*-axis. The monolayer B_4_N_4_ is composed of four- and eight-membered rings. The four-membered rings have bond lengths of 1.48 Å and bond angles of 84.26° and 95.74°. The eight-membered rings consist of two different B-N bonds with bond lengths of 1.48 and 1.40 Å and bond angles of 137.62° and 132.47°, respectively. B atoms occupy the 4e sites while N atoms prefer to occupy the 6c sites. Due to the weak van der Waals force between two neighboring atomic layers of bulk B_4_N_4_ along the *c*-axis, it is easy for them to form monolayers (Fig. 1(b)). The optimized lattice constants of monolayer B_4_N_4_ are *a* = *b* = 4.9422 Å and *γ* = 90°.

**Figure 2:**
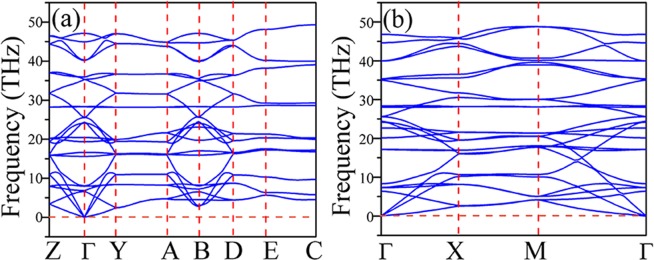
Phonon dispersion relations of (**a**) bulk B_4_N_4_ and (**b**) monolayer B_4_N_4_.

**Figure 3:**
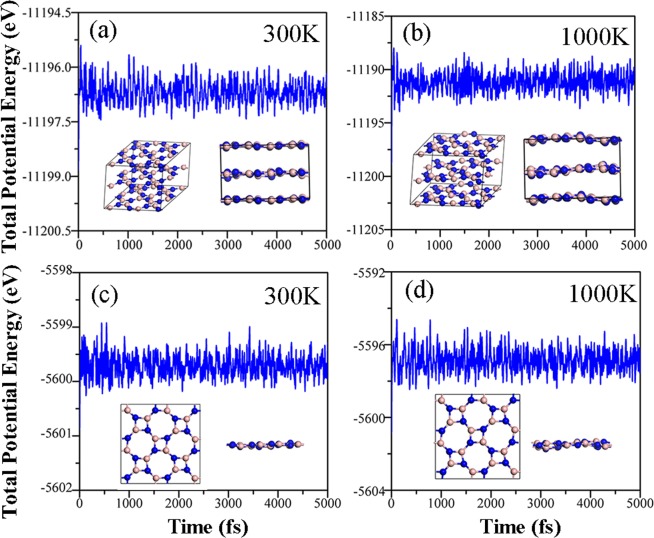
Time-dependent potential energy fluctuations of bulk B_4_N_4_ and monolayer B_4_N_4_ at 300 and 1000 K.

It is well known that phonon dispersion relations can reveal the structural stability of materials. As shown in Fig. 2, there is no imaginary frequency to be found in the phonon spectra of bulk B_4_N_4_ and monolayer B_4_N_4_, indicating that they are dynamically stable. Both the phonon spectra of bulk B_4_N_4_ and monolayer B_4_N_4_ have 24 kinds of dispersion relations, including three acoustic branches and 21 optical branches, because there are respectively eight atoms in each unit cell of bulk B_4_N_4_ and monolayer B_4_N_4_. The time-dependent potential energy fluctuations within 5 ps in MD simulations of bulk B_4_N_4_ and monolayer B_4_N_4_ plotted in Fig. 3 further verify that they are thermally stable over a wide temperature range. The geometrical structures of bulk B_4_N_4_ and monolayer B_4_N_4_ have changed to some extent, and the deformation degree at high temperature is greater than that at room temperature. As a whole, bulk B_4_N_4_ and monolayer B_4_N_4_ have very small potential energy fluctuations throughout the MD simulations, suggesting that they can maintain their stable crystal structures at 300 K, even at 1000 K.

### Mechanical properties

The unique crystal structures of bulk B_4_N_4_ and monolayer B_4_N_4_ would bring interesting mechanical properties under principal and/or shear strains. For the monoclinic B_4_N_4_ system, there are 13 independent elastic constants, *C*_11_, *C*_22_, *C*_33_, *C*_44_, *C*_55_, *C*_66_, *C*_12_, *C*_13_, *C*_14_, *C*_23_, *C*_24_, *C*_34_ and *C*_56_ (in unit of GPa) in the 6 × 6 stiffness tensor matrix, and they are written as:

1 $${C}_{ij}=(\begin{array}{cccccc}573.36 & 353.33 & -2.73 & 2.22\, & 0 & 0\\ 353.33 & 568.83 & -2.09 & -2.78 & 0 & 0\\ -2.73 & -2.09 & 34.52 & -0.57 & 0 & 0\\ 0 & 0 & 0 & 5.88 & 0 & 0\\ 2.22 & -2.78 & -0.57 & 0 & 1.51 & 0.96\\ 0 & 0 & 0 & 0 & 0.96 & 318.86\end{array})$$

For the two-dimensional monolayer B_4_N_4_, the calculated independent elastic constants, *C*_11_, *C*_12_, *C*_22_ and *C*_66_ (in unit of N/m), in the stiffness tensor matrix are:

2 $${C}_{ij}=(\begin{array}{c}185.42\\ 115.06\\ \vdots \\ 0\end{array}\begin{array}{c}\,115.06\,\\ \,185.42\\ \vdots \\ \,0\end{array}\begin{array}{cc}\,\cdots  & 0\\ \,\cdots  & 0\\ \,\ddots  & \vdots \\ \,\cdots  & \,104.06\end{array})$$

According to the Born-Huang criterion^21^, the mechanical stability of a monoclinic system satisfies:

$${C}_{11} > 0,{C}_{22} > 0,{C}_{33} > 0,{C}_{44} > 0,{C}_{55} > 0,{C}_{66} > 0,$$

$$({C}_{33}{C}_{55}-{C}_{35}^{2}) > 0,({C}_{44}{C}_{66}-{C}_{46}^{2}) > 0,({C}_{22}\,+{C}_{33}-2{C}_{23}) > 0,$$

3 $$[{C}_{11}+{C}_{22}+{C}_{33}+2({C}_{12}+{C}_{13}+{C}_{23})] > 0$$

For a two-dimensional monolayer, its structural mechanical stability requires^21^:

4 $${C}_{11}{C}_{22}-{C}_{12}^{2} > 0,\,{C}_{66} > 0$$

It is obvious that the elastic constants of bulk B_4_N_4_ satisfy the conditions mentioned in Eq. (3), suggesting that bulk B_4_N_4_ is mechanically stable under elastic strain perturbations. Due to the weak van der Waals force parallel to the *z* direction, *C*_33_ is much smaller than *C*_11_ and *C*_22_, indicating that the *z* direction of bulk B_4_N_4_ is easier to deform than the *x* and *y* directions. At the same time, *C*_66_ is much larger than *C*_44_ and *C*_55_, meaning that the *xy* plane has higher ability to resist shear deformation than the *xz* and *yz* planes. From *C*_*ij*_ values of monolayer B_4_N_4_ and Eq. (4), we know that monolayer B_4_N_4_ is also mechanically stable. Other elastic parameters of bulk B_4_N_4_ and monolayer B_4_N_4_, such as the bulk moduli (*B*), shear moduli (*G*), elastic moduli (*E*) and Poisson’s ratios (*v*), calculated by the Voigt-Ruess-Hill method^22–24^ are respectively shown in Tables 1 and 2. Particularly, similar to the bulk modulus (*B*), the layer modulus (*γ*) in Table 2 represents the ability of two-dimensional materials to resist plane strains.

**Table 1:** The calculated bulk modulus (*B*, GPa), shear modulus (*G*, GPa), Young’s modulus (*E*, GPa) and Poisson’s ratio (*v*) of bulk B_4_N_4_.

	*B*	*G*	*E*_x_	*E*_y_	*E*_z_	*E*	*v*
bulk B_4_N_4_	119.97	63.05	351.19	348.13	34.46	160.96	0.27

**Table 2:** The calculated layer modulus (*γ*, N/m), shear modulus (*G*, N/m), Young’s modulus (*E*, N/m) and Poisson’s ratio (*v*) of monolayer B_4_N_4_.

	*γ*	*G*	*E*_x_	*E*_y_	*E*	*v*
monolayer B_4_N_4_	150.24	104.06	114.02	114.02	114.02	0.62

From Table 1, we can see that the Young’s moduli of bulk B_4_N_4_ vary greatly along different directions, indicating that bulk B_4_N_4_ has strong anisotropy. *E*_z_ = 34.46 GPa, which is much smaller than *E*_x_ = 351.19 GPa and *E*_y_ = 348.13 GPa, showing the low strength of bulk B_4_N_4_ along the *z* direction because of the weak van der Waals force. In Table 1, the Young’s moduli in the *x* and *y* directions are very close, and the *xy* plane has high strength. We also calculated the universal anisotropy index (*A*^U^) expressed by the following formula^25^:

5 $${A}^{{\rm{U}}}=5\frac{{G}_{{\rm{V}}}}{{G}_{{\rm{R}}}}+\frac{{B}_{{\rm{V}}}}{{B}_{{\rm{R}}}}-6\ge 0$$

The calculated *A*^U^ = 107.37 indicates that bulk B_4_N_4_ has strong anisotropy, which is the manifestation that the components of Young’s moduli vary greatly in different directions. The Young’s moduli of monolayer B_4_N_4_ in the *x* and *y* directions are equal due to the square symmetric structures. Besides, monolayer B_4_N_4_ has a special large Poisson’s ratio of 0.62, and it should be attributed to the loose arrangements of atoms allowing greater contractions during deformation^26^.

### Electronic structures

**Figure 4:**
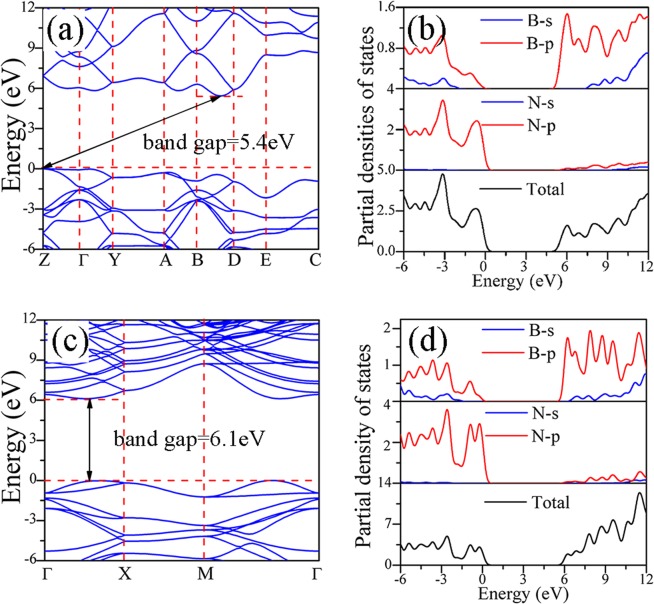
Band structures and partial densities of states of (**a**,**b**) bulk B_4_N_4_ and (**c**,**d**) monolayer B_4_N_4_.

Figure 4 shows the band structures and partial densities of states of bulk B_4_N_4_ and monolayer B_4_N_4_ in the first Brillouin zone. The Fermi level is set to zero. Bulk B_4_N_4_ has an indirect band gap of 5.4 eV, while monolayer B_4_N_4_ has a direct band gap of 6.1 eV. In Table 3, the band gaps of c-BN and h-BN calculated by the hybrid functional PBE0 are in good agreement with the experimental values^27–29^, so we can deduce that PBE0 is suitable to estimate the band gaps of bulk B_4_N_4_ and monolayer B_4_N_4_. The obtained band gap of monolayer B_4_N_4_ is close to those of c-BN and h-BN, and the band gap of bulk B_4_N_4_ is slightly smaller than those of c-BN, h-BN and B_4_N_4_ nanosheet. From the partial densities of states of bulk B_4_N_4_ and monolayer B_4_N_4_, the valence band maximum (VBM) is mainly contributed from B-2*p* and N-2*p* hybrid orbits, and the conduction band minimum (CBM) mainly derives from B-2*p* orbits.

**Table 3:** Band gaps of bulk B_4_N_4_, monolayer B_4_N_4_, c-BN, and h-BN.

	GGA-PBE	PBE0	Exp.
bulk B_4_N_4_	3.5	5.4	/
monolayer B_4_N_4_	4.1	6.1	/
c-BN	4.5	6.5	6.0^a^, 6.4^b^
h-BN	4.4	6.2	5.9^c^, 5.8^d^

^a,b^Ref. ^28^, ^c^ref. ^29^ and ^d^ref. ^27^

### Optical properties

**Figure 5:**
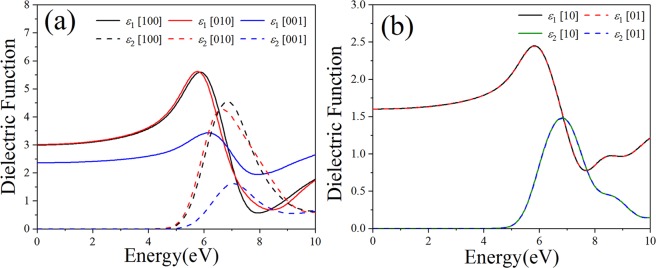
The dielectric functions of (**a,c**) bulk B_4_N_4_ and (**b,d**) monolayer B_4_N_4_.

The optical properties of some a medium can be described by the complex dielectric function: *ε*(ω) = *ε*_1_(ω) + i*ε*_2_(ω)^30,31^. The real part *ε*_1_(*ω*) characterizes the polarization degree of medium under an external electric field. The imaginary part *ε*_2_(*ω*) corresponding to the interband electron transitions from occupied states to unoccupied states can be obtained by calculating the momentum matrix elements of the wave function, and then *ε*_1_(*ω*) can be obtained from the Kramers–Kronig correlation^32,33^. Because bulk B_4_N_4_ belongs to the monoclinic system, it is necessary to consider the optical anisotropy of bulk B_4_N_4_ and its corresponding monolayer B_4_N_4_. Figure 5 shows the direction-dependent dielectric functions of bulk B_4_N_4_ and monolayer B_4_N_4_ in the energy range of 0–10 eV. For bulk B_4_N_4_, see Fig. 5(a), the photon energy-dependent intensities and peak positions of dielectric functions in [100] and [010] directions are slightly different, while those in [001] direction are quietly different, which should be attributed to the influence of van der Waals force in [001] direction, see Fig. 1(a). The calculated static dielectric constants ${\varepsilon }_{1}^{[100]}(0)$, ${\varepsilon }_{1}^{[010]}(0)$ and ${\varepsilon }_{1}^{[001]}(0)$ of bulk B_4_N_4_ are respectively 2.99, 3.01 and 2.36, and there are three major peaks locating at 5.88, 5.76 and 6.18 eV in ${\varepsilon }_{1}^{[100]}(\omega )$, ${\varepsilon }_{1}^{[010]}(\omega )$ and ${\varepsilon }_{1}^{[010]}(\omega )$, respectively. In *ε*_2_(*ω*) of bulk B_4_N_4_, there exist three major peaks locating at 6.84, 6.64 and 7.06 eV respectively corresponding to ${\varepsilon }_{2}^{[100]}(\omega )$, ${\varepsilon }_{2}^{[010]}(\omega )$ and ${\varepsilon }_{2}^{[001]}(\omega )$, which can be attributed to the electron transitions from N-2*p* to B-2*p* states. As regards to monolayer B_4_N_4_ in Fig. 5(b), however, the dielectric functions in [10] and [01] directions are completely the same, implying the influence of van der Waals force disappears. The theoretical static dielectric constants ${\varepsilon }_{1}^{[10]}(0)$ and ${\varepsilon }_{1}^{[01]}(0)$ are 1.59, and the same peak in ${\varepsilon }_{1}^{[10]}(\omega )$ and ${\varepsilon }_{1}^{[01]}(\omega )$ locates at 5.82 eV. ${\varepsilon }_{2}^{[10]}(\omega )$ and ${\varepsilon }_{2}^{[01]}(\omega )$ with their main peak sited at 6.79 eV originate from the electron transitions from N-2*p* to B-2*p* states.

**Figure 6:**
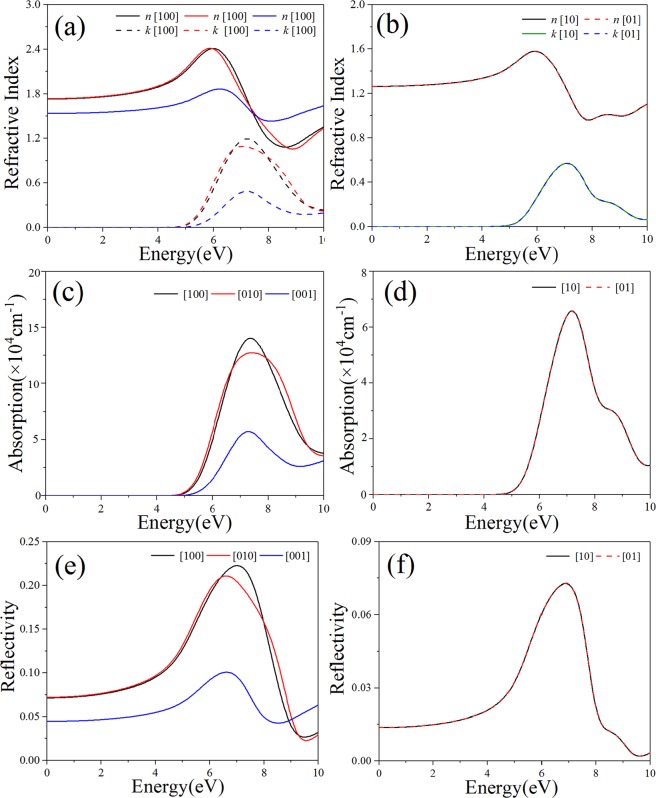
The refractive indexes, absorption coefficients and reflectivities of (**a,c,e**) bulk B_4_N_4_ and (**b,d,f**) monolayer B_4_N_4_.

The complex refractive index can be expressed as $\tilde{n}(\omega )=n(\omega )+{\rm{i}}k(\omega )$, where the refractive index *n*(ω) has the same trend as the real part of dielectric function, while the extinction coefficient *k*(ω) is similar to the imaginary part of dielectric function. The refractive indexes and extinction coefficients of bulk B_4_N_4_ and monolayer B_4_N_4_ are respectively displayed in Fig. 6(a,b). The static refractive indexes *n*(0) of bulk B_4_N_4_ are respectively 1.73, 1.74 and 1.54 along [100], [010] and [001] directions. For monolayer B_4_N_4_, in [10] and [01] directions, the static refractive indexes *n*(0) are 1.26. For bulk B_4_N_4_, the maximum refractive indexes of [100], [010] and [001] directions are respectively 2.42, 2.42 and 1.87 at the photon energies of 5.91, 6.00 and 6.24 eV. For monolayer B_4_N_4_, the energy-dependent refractive indexes in [10] and [01] directions reach the same maximum value of 1.58 at 5.91 eV. The extinction coefficients of bulk B_4_N_4_ and monolayer B_4_N_4_ are zero in the visible region. However, in the ultraviolet (UV) region, the extinction coefficients of bulk B_4_N_4_ have three peaks at 7.21, 7.06 and 7.18 eV respectively corresponding to [100], [010] and [001] directions, and those of monolayer B_4_N_4_ have the same peak at 7.07 eV in [10] and [01] directions.

The calculated absorption coefficients of bulk B_4_N_4_ and monolayer B_4_N_4_ are respectively shown in Fig. 6(c,d). The initial values of the first peak in the absorption spectra of bulk B_4_N_4_ and monolayer B_4_N_4_ are respectively 4.66 and 4.81 eV, which are significantly close to their band gaps. In the energy range of 0–10 eV, the absorption spectra of bulk B_4_N_4_ in [100], [010] and [001] directions respectively have their main peaks at 7.37, 7.43 and 7.28 eV, and those of monolayer B_4_N_4_ in [10] and [01] directions respectively have their main peaks at 7.17 eV. When the photon energy is greater than ~4.71 eV, bulk B_4_N_4_ and monolayer B_4_N_4_ begin to absorb incident light mainly in the ultraviolet region. In the considered energy region, bulk B_4_N_4_ has the highest absorption coefficient of 14.09 × 10^4^ cm^−1^ at 7.37 eV in [100] direction. In the photon energy region of ~5–9 eV, monolayer B_4_N_4_ can run up to the highest absorption coefficient of 6.59 × 10^4^ cm^−1^. As a whole, the absorption coefficient of monolayer B_4_N_4_ is lower than that of bulk B_4_N_4_ in the energy region of 0–10 eV.

In the visible region, see Fig. 6(e,f), the reflectivities of bulk B_4_N_4_ in [100] and [010] directions ([001] direction) are about ~7.7% (~4.7%), and those of monolayer B_4_N_4_ in [10] and [01] directions are only ~1.5%. In the ultraviolet region, the highest reflectivity of bulk B_4_N_4_ is ~22.29% in [100] direction, while that of monolayer B_4_N_4_ is ~7.30%. The reflectivity of monolayer B_4_N_4_ is much lower than that of bulk B_4_N_4_. Consequently, monolayer B_4_N_4_ has a high transmissivity due to its low absorption and reflectivity, and it has potential applications in optoelectronic devices such as transparent electrodes.

**Figure 7:**
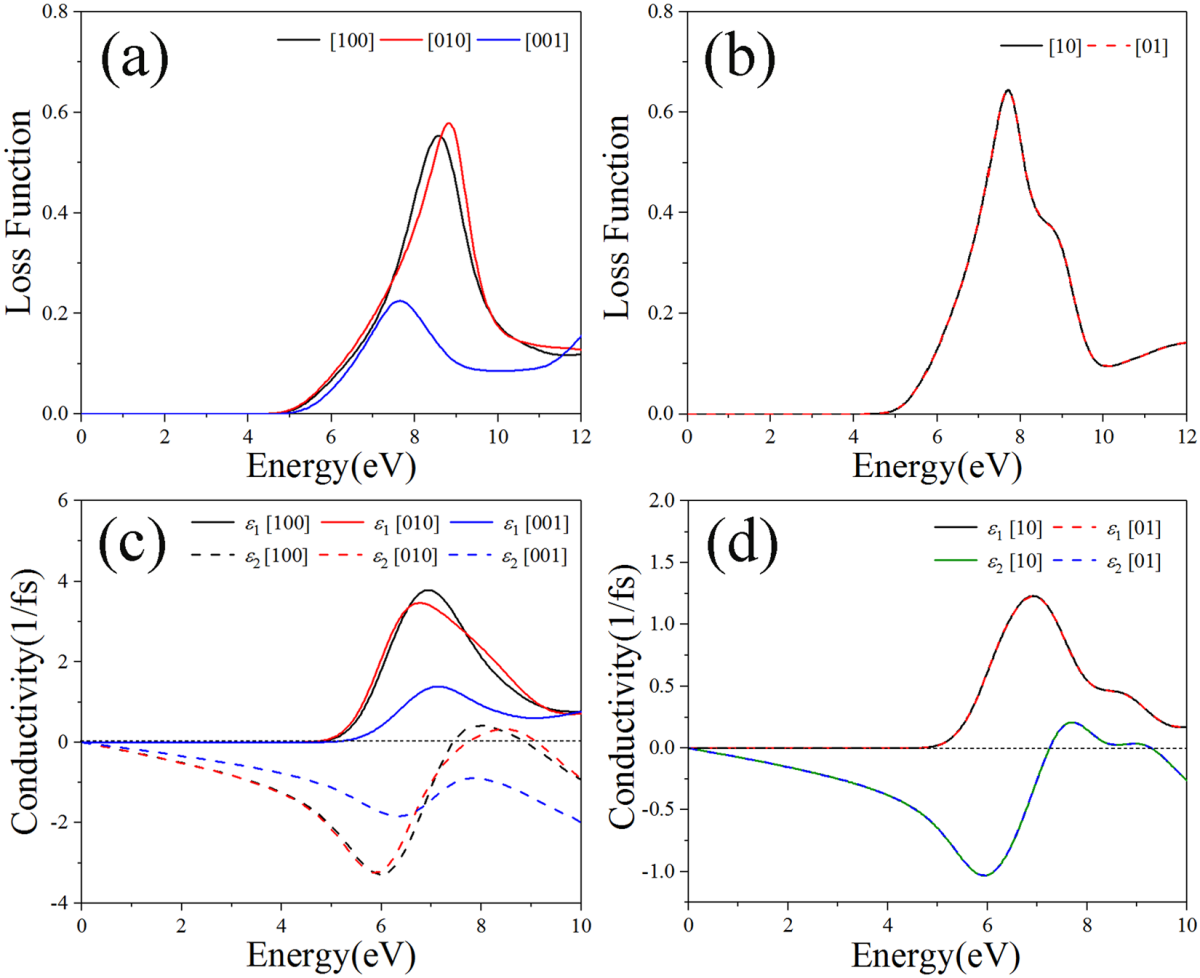
The loss functions and conductivities of (**a,c**) bulk B_4_N_4_ and (**b,d**) monolayer B_4_N_4_.

Loss function depicts the energy loss of photoelectrons when they pass through uniform dielectrics, and the position of characteristic peak in loss function generally corresponds to the plasma frequency of dielectrics^34^. The loss functions of bulk B_4_N_4_ and monolayer B_4_N_4_ are respectively plotted in Fig. 7(a,b), and one can see that the energy loss of photoelectrons occurs in the energy region greater than ~4.85 eV. The real and imaginary parts of the conductivities shown in Fig. 7(c,d) indicate that both bulk B_4_N_4_ and monolayer B_4_N_4_ have high conductivities in the energy range of ~6–8 eV and get their maximum conductivities at ~7 eV.

## Conclusions

In the current work, we proposed bulk B_4_N_4_ and monolayer B_4_N_4_ and investigated their phonon dispersion relations, mechanical properties, electronic structures and optical properties by first-principles based on density functional theory. The crystal structure of bulk B_4_N_4_ consists of monolayer B_4_N_4_ connected by the van der Waals force, and the monolayer B_4_N_4_ is composed of four- and eight-membered rings. Bulk B_4_N_4_ and monolayer B_4_N_4_ are dynamically, thermally and mechanically stable. The elastic constants show that bulk B_4_N_4_ has large anisotropy. Moreover, through prediction of the PBE0 method, bulk B_4_N_4_ has an indirect band gap of 5.4 eV while monolayer B_4_N_4_ has a direct band gap of 6.1 eV. In either bulk B_4_N_4_ and monolayer B_4_N_4_, the VBM is mainly contributed from B-2*p* and N-2*p* orbits and the CBM mainly derives from B-2*p* states. Monolayer B_4_N_4_ with a high transmissivity has great potential in designing new optoelectronic devices such as transparent electrodes.
